# 4332 Septic Shock Epidemiology and Sociodemographic Predictors of Mortality: Results from One Florida Data Trust Cohort

**DOI:** 10.1017/cts.2020.145

**Published:** 2020-07-29

**Authors:** Lauren Page Black, Charlotte Hopson, Elizabeth DeVos, Rosemarie Fernandez, Faheem Guirgis, Cynthia Garvan

**Affiliations:** 1 University Of Florida Clinical and Translational Science Institute; 2 University of Florida College of Medicine - Jacksonville; 3 University of Florida College of Medicine; 4 University of Florida

## Abstract

OBJECTIVES/GOALS: Septic shock is a lethal condition. Research suggests that overall sepsis mortality varies by race, but less is known about demographic differences in septic shock mortality. Our objectives were to describe the septic shock population using a large, state-wide data repository and identify demographic predictors of septic shock mortality. METHODS/STUDY POPULATION: This was a retrospective review of patients with septic shock in the One Florida Data Trust from 2012-2018. Patients were classified as having septic shock if they received vasopressors and had either 1) an ICD-9 or 10 code for septic shock or 2) an ICD-9 or 10 code for infection and an ICD-9 or 10 code for organ dysfunction. Demographic data and place of residence prior to admission was collected. The primary outcome was 90 day mortality. T-test and chi-square tests were used to test association of individual predictors and mortality. Multiple logistic regression was used to identify predictors of mortality after adjustment for other variables. Level of significance was set at 0.05. SAS v9.4 (Cary, NC) was used for analyses. RESULTS/ANTICIPATED RESULTS: There were 11,790 patients with septic shock. The mean(SD) age was 61(16) years. With regard to race/ethnicity 66% identified as white, 27% as black, 3.7% as Hispanic, and 3.5% as other races (non-white, non-black, non-Hispanic). Most came from home (57%). Overall, 39% died. Mortality varied by race (p<0.01): white 39%, black 39%, Hispanic 31%, other races 51%. In the logistic regression model, age, race, and residence were significant predictors of mortality, after adjustment for other variables. Each additional year of age had a 2.7% increased odds of mortality (OR 1.03; 95% CI 1.02-1.03; p<0.01). Compared to white patients, odds of death were 1.6 times higher for other races (95% CI 1.3-2.0; p <0.01) and non-significantly higher for black patients (OR 1.1; 95% CI 1.0-1.2; p = 0.05). Compared to those from home, odds of death were highest for those from a skilled nursing facility (OR 1.5; p<0.01). DISCUSSION/SIGNIFICANCE OF IMPACT: Patients who identified as other races had increased mortality from septic shock compared to white patients after adjusting for other variables. Septic shock mortality also increased with age and varied by residence. Further analyses are needed to examine racial disparities and control for comorbidities, severity of illness, and aspects of resuscitation. CONFLICT OF INTEREST DESCRIPTION: The authors report no conflicts of interest, except for Dr. Fernandez, who reports personal payment from Physio-Control, Inc. for speaker fees.

